# Influence of Elastic Stiffness and Surface Adhesion on Bouncing of Nanoparticles

**DOI:** 10.1186/s11671-017-2410-4

**Published:** 2017-12-22

**Authors:** Philipp Umstätter, Herbert M. Urbassek

**Affiliations:** Physics Department and Research Center OPTIMAS, University Kaiserslautern, Erwin-Schrödinger-Straße, Kaiserslautern, D-67663 Germany

**Keywords:** Granular mechanics, Grain collisions, Morse potential, Johnson-Kendall-Roberts theory, Atomistic simulation

## Abstract

Granular collisions are characterized by a threshold velocity, separating the low-velocity regime of grain sticking from the high-velocity regime of grain bouncing: the bouncing velocity, *v*_*b*_. This parameter is particularly important for nanograins and has applications for instance in astrophysics where it enters the description of collisional dust aggregation. Analytic estimates are based on the macroscopic Johnson-Kendall-Roberts (JKR) theory, which predicts the dependence of *v*_*b*_ on the radius, elastic stiffness, and surface adhesion of grains. Here, we perform atomistic simulations with model potentials that allow us to test these dependencies for nanograin collisions. Our results not only show that JKR describes the dependence on materials parameters qualitatively well, but also point at considerable quantitative deviations. These are the most pronounced for small adhesion, where elastic stiffness does not influence the value of the bouncing velocity.

## Background

Arguably, the most basic process of granular mechanics is the collision of two grains. At large grain velocities, grains separate again after the collision, and the collision outcome can be characterized by the classical mechanics of inelastic collisions. At small grain velocities, however, grains will stick. The boundary between sticking and bouncing collisions [1] may be termed the bouncing velocity, *v*_*b*_. This parameter is particularly important for nanograins and has applications for instance in astrophysics where it enters the description of collisional dust aggregation [2, 3].

Macroscopic contact mechanics has been used to derive a prediction for *v*_*b*_. It is based on the Johnson-Kendall-Roberts (JKR) theory [4], which describes the collision of two adhesive spheres using the elastic stiffness and the surface adhesion as basic physics input. Quantitatively, these quantities are described by the indentation modulus, *E*_ind_=*E*/(1−*ν* ^2^), where *E* is the Young modulus and *ν* the Poisson number, and by the surface energy *γ*. With the sphere radius *R* and the mass density *ρ*, the bouncing velocity of two identical spheres reads [1, 5, 6]

1 $$ {v_{b}} = \left(\frac {C} {\rho} \right)^{1/2}\left(\frac {\gamma^{5}} {E_{\text{ind}}^{2} R^{5}} \right)^{1/6}.  $$

The value of the constant *C* depends strongly on the assumptions of energy dissipation during the collision and has been discussed to assume values between 0.3 and 60 [1, 7].

The validity of this prediction has been predominantly studied with respect to its size dependence [1, 5–8]. With decreasing grain size, adhesive forces become more important, and the bouncing velocity increases. Indeed, experiments on nanograins (Ag and NaCl grains) [9] find *v*_*b*_ to be in the range of 1 m/s for grain sizes of a few 10 nm, but to increase sharply for smaller grains. Atomistic simulations based on molecular dynamics (MD) have confirmed the predicted *R* ^−5/6^ dependence for collisions between amorphous silica grains of sizes *R*=15–25 nm [7].

Up to now, the predicted dependence of *v*_*b*_ on the materials parameters *E*_ind_ and *γ* has not been tested in detail. This is not easily done in experiment, since different materials differ usually in both quantities. However, using MD, we can construct model materials, which have identical properties, but differ only in one aspect, either *E*_ind_ or *γ*. In this paper, we choose a model for Cu [10] but vary the materials parameters generously by up to one order of magnitude from the real values. Since we find no bouncing for amorphous nanoparticles in this system, we focus on crystalline (fcc) grains.

## Methods/Experimental

We use the Morse potential,

2 $$ U(r) = D \left[ e^{-2\alpha(r-r_{0})} - 2 e^{-\alpha(r-r_{0})} \right],  $$

to describe the interaction between two atoms of distance *r*. The three Morse parameters *D*, *α*, and *r*_0_ are determined to describe the lattice constant *a*, the bulk modulus *B*, and the cohesive energy *E*_coh_ of a bulk fcc solid.

For definiteness, we fix the lattice constant to *a*=3.615 Å (appropriate for Cu) in this study and also adopt the atomic mass of Cu, in order to keep the mass density *ρ* in Eq. (1) fixed. The potential is cut off at *r*_*c*_=2.5*a*; thus, 12 neighbor shells, including a total of 248 atoms, interact with each atom. A number of 100 potentials are evaluated for *B* in the range of 403 to 1008 GPa, and *E*_coh_ in the range of 0.35 to 3.54 eV. Note that the bulk moduli studied here are larger, and the cohesive energies are smaller, than the values of real Cu (*B*=134.4 GPa, *E*_coh_=3.54 eV [11]), since for the real values, we did not observe any bouncing.

**Fig. 1:**
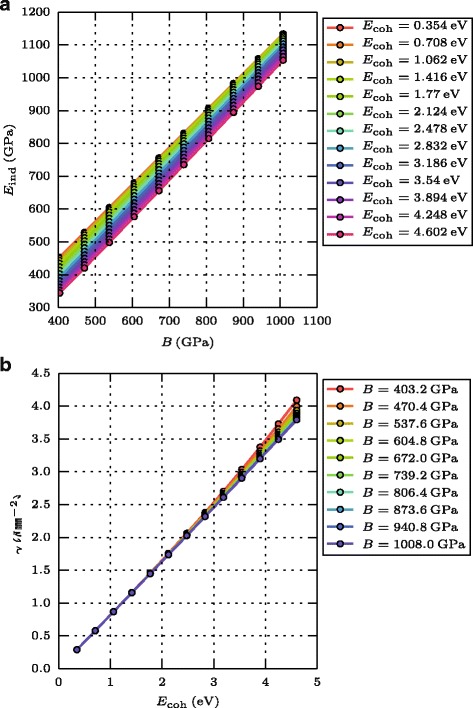
Materials parameters. Dependence of **a** the indentation modulus *E*_ind_ on the bulk modulus *B* and of **b** the surface energy *γ* on the cohesive energy *E*_coh_

We determine the indentation modulus *E*_ind_ for uniaxial stress in (100) direction from the Young modulus and the Poisson number in this direction ([12], p. 32). Figure 1 a displays the dependence of *E*_ind_ on *B*. We see that these quantities obey a linear relationship; at constant bulk modulus, a decrease of the cohesive energy lets *E*_ind_ increase.

The surface energy of (100) facets is calculated from the energy difference of a bulk crystal and a crystal with an open (100) surface by dividing through the area of the open surface [13]. Figure 1 b shows that *γ* is roughly proportional to *E*_coh_; deviations are only visible for smaller stiffnesses and strongly bonded materials.

**Fig. 2:**
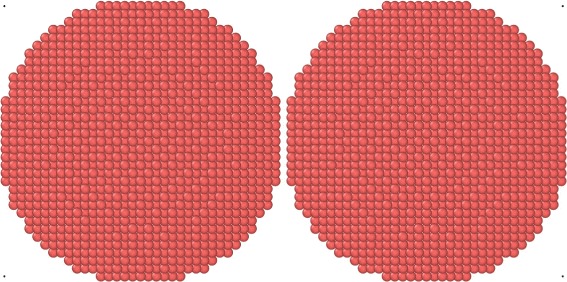
Initial setup of the collision

We construct grains by cutting a sphere with radius *R*=9*a*=33 Å out of the fcc lattice, containing around 12,000 atoms. Due to their construction, they have a facetted surface. They are relaxed in order to equilibrate their surfaces; slight surface relaxation, but no reconstruction of the surface was observed. The collisions are started by duplicating the grains and shooting them towards each other with a relative velocity *v*. Only central collisions are considered, where the two facing (100) facets collide head-on, see Fig. 2.

For determining the bouncing velocity, we perform collisions with several velocities. The algorithm used here is based on a simple bisection scheme. We verified that collisions with a velocity of 250 m/s are bouncing for all collision systems studied here, while at vanishing velocity, collisions are sticking. Then, simulations are run at the arithmetic mean of the lowest known bouncing velocity and the highest known sticking velocity. This procedure is repeated until the difference between the highest sticking and the lowest bouncing velocity is less than 10% of their mean value. *v*_*b*_ is taken as the arithmetic mean of the highest sticking velocity and the lowest bouncing velocity; these two latter values are also taken to indicate the error of our computation in the plots. The simulations were performed using the open-source software LAMMPS [14], and the code is essentially the same as that used in our previous studies on collisions of silica [7] and water-ice particles [15].

## Results

**Fig. 3:**
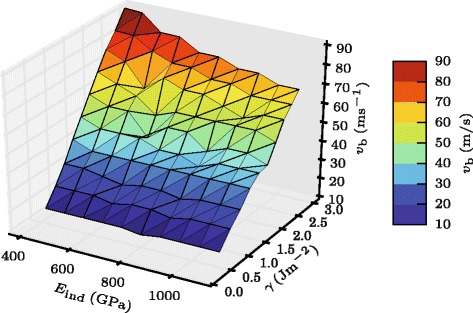
Bouncing velocity. Three-dimensional plot of the dependence of the bouncing velocity *v*_*b*_ on the indentation modulus *E*_ind_ and the surface energy *γ*

Figure 3 gives an overview over the results obtained. An overall power-law fit is provided by

3 $$ {v_{b}} \propto \gamma^{0.588} E_{\text{ind}}^{-0.155}.  $$

Thus, the main characteristics of the JKR law, Eq. (1)—an increase of *v*_*b*_ with adhesion and a decrease with elastic stiffness—are reproduced, but the dependencies are weaker than those in the JKR case.

**Fig. 4:**
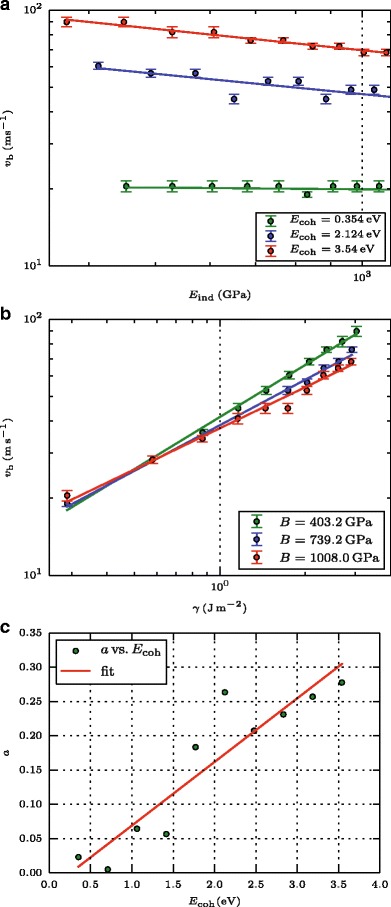
Bouncing velocity. Dependence of the bouncing velocity *v*_*b*_ on the **a** indentation modulus *E*_ind_ and the **b** surface energy *γ*. Lines denote power-law fits. **c** displays the dependence of the power exponent, *a*, Eq. (4), on the cohesive energy. The line denotes a linear fit to guide the eye

Figure 4 looks in more detail into these dependencies. Since we determined the bouncing velocities for materials with either fixed *B* or *E*_coh_, we will analyze them for these fixed values, but present the dependencies in terms of *E*_ind_ and *γ* in order to make connection with the JKR prediction, Eq. (1). For constant cohesive energy *E*_coh_, *v*_*b*_ depends like a power law on the elastic stiffness,

4 $$ v_{b} \propto E_{\text{ind}}^{-a},  $$

where *a*=0.28 (0.26, 0.02) for *E*_coh_=3.54 (2.12, 0.35) eV. Thus, the exponent *a*=0.33 predicted by JKR is indeed nearly recovered for high surface energies; however, the dependence becomes softer with decreasing *γ* and vanishes altogether for weakly adhesive surfaces. Note that in the case of vanishing surface energy, all collisions must be bouncing; this explains the vanishing role of the elastic stiffness in this case.

Figure 4 c displays the power exponents of the dependence of *v*_*b*_(*E*_ind_), Eq. (4), obtained from our simulations. The plot clearly demonstrates the increase of the dependence on *E*_ind_ with increasing cohesive energy, and hence surface energy, as indicated by the red linear fit line.

For fixed elastic stiffness, *B*, the dependence of *v*_*b*_ on *γ* shows a simpler picture, see Fig. 4 c. Power-law fits, *v*_*b*_∝*γ* ^−*b*^, give rather consistent values of *b*=0.67 (0.59, 0.53) for *B*=403 (739, 1008) GPa, and thus show only a mild dependence on *B* and hence *E*_ind_. Note, however, that these dependencies are softer than the value of *b*=0.83 predicted by Eq. (1). With increasing stiffness, the deviations from the JKR prediction become stronger. Indeed, it is known that JKR fails for too stiff systems [16, 17]. For such systems, the Derjaguin-Muller-Toporov (DMT) theory [18] is thought to apply better; however, no prediction for the bouncing velocity seems to have emerged from that theory.

Overall, the bouncing velocities found here are below 100 m/s. We emphasize that for realistic values of the Morse potential as appropriate for Cu, we find sticking over the entire range of velocities, and no bouncing. This is in line with recent simulations of Cu sphere (7–22 nm diameter) collisions with an Al surface performed by Pogorelko et al. [19, 20] who find sticking up to velocities of 1000 m/s. The reason we do find bouncing in our simulations is that we use model potentials in which the elastic moduli are generously increased, and the surface binding is decreased, with respect to the values characterizing real Cu.

Above the bouncing threshold, collisions are characterized by the coefficient of restitution,

5 $$ e= |v'|/|v|,  $$

which compares the relative velocity after collision, *v* ^′^, to that before the collision, *v*, and thus measures the inelasticity of the collision. For sticking collisions, evidently, *e*=0. JKR theory suggests a law [4–6]

6 $$ e_{\text{JKR}} = \alpha \sqrt{1- \left(\frac{v_{b}}{v} \right)^{2}},  $$

where we introduced the factor *α* to take energy dissipation into account [7].

**Fig. 5:**
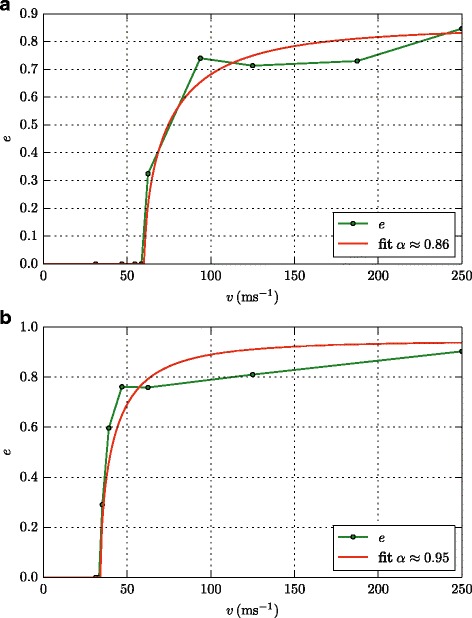
Coefficient of restitution. Dependence of the coefficient of restitution, *e*, on collision velocity, *v*, for a strongly (*γ*=2.32 J/m^2^) (**a**) and a weakly (*γ*=0.89 J/m^2^) (**b**) adhesive surface. The bulk modulus is identical in both cases, *B*=940.8. Symbols denote simulation results, while the curve is a fit to the JKR prediction, Eq. (6), with *α*=0.86 (**a**) and 0.95 (**b**)

Figure 5 displays two cases of the velocity dependence of *e*; we find these to be representative for the entire range of stiffness and adhesion values investigated. In all these cases, there is no major energy dissipation during the collision; *α* is around 0.9. At sufficiently large surface energies, Fig. 5 a, *e* follows quite well the JKR prediction, Eq. (6). At small *γ*, however, Fig. 5 b, a narrower transition zone is seen, in which *e* switches from 0 to almost 1; this transition zone is not well described by the JKR prediction, Eq. (6).

## Discussion

In the sticking regime, the coefficient of restitution stays below 1 indicating inelastic energy losses during the collision. We verified that the collisions are purely elastic in the sense that no permanent plasticity was generated during the collision; the software tool OVITO [21] was used to check for dislocation production. For higher velocities, *v*>100 m/s, and compliant spheres, dislocations were formed transiently but disappeared again after the collision. We note that during the collision of similarly sized crystalline nanospheres interacting via the generic Lennard-Jones potential, ample dislocation production could be detected [22, 23], while shear transformation zones were identified in the collision of amorphous silica spheres [7], both collision systems thus exhibit plasticity. In our case, the high elastic moduli prevent the establishment of plastic deformation; inelastic energy losses are caused only by the excitation of vibrations in the collided spheres. It may be concluded that the existence of bouncing collisions is connected to a suppression of inelastic losses during the collisions and thus to the suppression of plastic deformation.

The behavior of *e* for small *γ* underlines our above findings for *v*_*b*_ that large deviations from JKR are exhibited for weakly adhesive systems. We conclude that for weak adhesion, the bouncing velocity, and also the state of the system after bouncing depend only weakly on other system characteristics, such as *E*_ind_ and *v*.

## Conclusions

The prediction of the JKR theory of adhesive elastic contacts has been tested by dedicated MD simulations of nanograins using model potentials. We find that the gross trends of the dependence of the bouncing velocity are reasonably well reproduced by JKR theory when varying the material stiffness and the material adhesion by up to an order of magnitude. However, we find systematic deviations for weakly adhesive grains; in this case, the bouncing threshold becomes independent of the material stiffness, and the coefficient of restitution exhibits almost no velocity dependence above *v*_*b*_. Also for stronger adhesion, the dependence of the bouncing velocity on *γ* is systematically smaller than that predicted by JKR.

These deviations point at an incomplete description of nanoparticle collisions by macroscopic contact theory. Future work will attempt to extend this study to crystalline grains with other orientations and with larger radii, and to amorphous grains.
